# Effects of COVID-Induced Public Anxiety on European Stock Markets: Evidence From a Fear-Based Algorithmic Trading System

**DOI:** 10.3389/fpsyg.2021.780992

**Published:** 2022-01-14

**Authors:** Yunpeng Sun, Haoning Li, Yuning Cao

**Affiliations:** School of Economics, Tianjin University of Commerce, Tianjin, China

**Keywords:** COVID-19, fear, stock market returns, Google Trends, Wikipedia, algorithmic system trading

## Abstract

The effect of COVID-induced public anxiety on stock markets, particularly in European stock market returns, is examined in this research. The search volumes for the notion of COVID-19 gathered by Google Trends and Wikipedia were used as proxies for COVID-induced public anxiety. COVID-induced public anxiety was shown to be linked with negative returns in European stock markets when a panel data method was used to a sample of data from 14 European stock markets from January 2, 2020 to September 17, 2020. Using an automated trading system, we used this finding to suggest investment methods based on COVID-induced anxiety. The findings of back-testing indicate that these techniques have the potential to generate exceptional profits. These results have significant consequences for government officials, the media, and investors.

## Introduction

The coronavirus disease (COVID-19) outbreak, which the WHO designated as a pandemic on March 11, 2020, has caused the most major shift in the world order since the previous century, creating economic problems on a scale not seen since the Great Depression of 1929 (Laing, 2020).

Governments all around the globe were confronted with significant difficulties, including a profound economic downturn, rising unemployment, a dramatic decrease in international commerce, and rising budget deficits (Bai et al., 2021). It did not take long for the COVID-19 effect to be felt in the stock market. The rising number of infections has prompted governments to take countermeasures, but it has also resulted in a catastrophic drop in stock markets, with several worldwide markets seeing their worst collapses in history in February and March 2020 (Li et al., 2021). For example, the S&P 500, a stock market benchmark index in the United States, had lost 35% of its value by March 23, 2020, compared to its then-historic high on February 19, 2020.

COVID-19 is the father of all worries that have impacted the world’s global financial and economic systems (Phan and Narayan, 2020). According to Aslam et al. (2020), the dread connected with the number of reported fatalities has resulted in a higher level of anxiety and a faster spread of panic than the virus itself. Fear in the stock market has been proven to be a mediator and a transmission route for the COVID-19 epidemic effect (Liu et al., 2020). Investors may overreact or underreact as a result of this anxiety (Daniel et al., 1998; Hong et al., 2007).

Therefore, the purpose of this article is to examine the effect of COVID-induced public anxiety on financial markets, particularly European stock market returns. When individuals are afraid, they tend to look for additional information about the circumstances that have caused them to feel this way (Chen et al., 2019). This study utilized Google Trends and Wikipedia search volumes for the COVID-19 as a foundation for assessing COVID-induced public anxiety. The anxiety generated by COVID-19, as assessed by Google Trends, had a negative and substantial effect on European stock market returns, according to a panel data methodology applied to a sample of 13 nations for the period January 2, 2020 to September 17, 2020. When delays were included in the explanatory factors, the finding was much more robust. Based on the findings, it was thought appropriate to suggest a trading technique to assess the potential to achieve exceptional advantages by integrating fear. Previous research on the impact of COVID-induced public anxiety on stock market returns (Salisu and Vo, 2020) and volatility (Lyócsa et al., 2020) has been conducted. During the COVID-19 epidemic, we utilized Google Trends’ forecasting abilities to build an algorithmic trading system. Our findings demonstrated that an algorithmic trading strategy based on COVID-induced public anxiety may generate exceptional gains in European stock markets. To the contribution, (1) we add to the literature on the impact of exogenous events and the ability of internet search volumes to measure the impact of public fear on financial markets; (2) we contribute to studies on the impact of COVID-19 on financial markets by using Google Trends and Wikipedia as indicators of COVID-induced public fear; (3) we show how COVID-induced public fear has a positive impact on financial markets.

This is how the article is organized. The theoretical foundation for the research is discussed in section “Conceptual Framework.” Section “Research Methodology” includes the data and suggested approach. The findings are presented and discussed in section “Results and Discussion.” Finally, in section “Conclusion,” the study’s findings are presented.

## Conceptual Framework

Since the 1918 Spanish Flu epidemic, the COVID-19 has been a significant worldwide danger to life and livelihood (Zhou et al., 2020). This time, the fast spread of the virus and high death rates sparked widespread public worry, resulting in anxiety and even panic, independent of geography or COVID-19 exposure (Nicomedes and Avila, 2020). Multiple studies on COVID-19’s impact on geopolitical risk (Sharif et al., 2020), the economy (Jordà et al., 2020; Mohsin et al., 2021), labor markets and employment (Coibion et al., 2020), large companies (Hassan et al., 2020), and small business (Fairlie, 2020) have been published in a short period of time. Studies by Al-Awadhi et al. (2020) and Liu et al. (2020) in financial markets revealed evidence of the rapid effect of verified COVID-19 cases and fatalities in these markets. Several researches have looked at the effect of COVID-19-induced negative emotion on stock market performance from a behavioral finance viewpoint. Bansal (2020) investigated the impact of cognitive mistakes and biases on financial institutions and markets during and after the COVID-19 crisis. Mann et al. (2020) looked at demographic and individual relationships for distress anxiety, the day after the COVID-19 pandemic announcement caused record stock market collapses. When individuals encounter financial problems, they found that age, location, children, self-esteem, awareness, openness to experience, neuroticism, susceptibility to disease, and group participation were deciding variables in anxiety levels. Zaremba et al. (2020) looked at the effect of government actions to limit the spread of COVID-19 on stock market volatility and found that non-pharmaceutical measures increased stock market volatility substantially. They discovered that this impact was independent of the COVID-19 pandemic’s involvement, and that it was resilient to a variety of factors. In response to COVID-19 pronouncements, Erdem (2020) investigated the potential connection between a country’s degree of freedom and stock performance, and discovered that the detrimental impacts of the COVID-19 on stock markets were smaller in the freest nations. Salisu et al. (2020a) investigated how developing stock markets react to the risk of pandemics and epidemics, such as the COVID-19 pandemic. Emerging stock markets, they discovered, are more susceptible to the unpredictability of pandemics and epidemics than established stock markets (Sun and Li, 2021). The COVID-19 danger came as a surprise, prompting individuals to look for additional information about the pandemic. People pay greater attention to the current news and significant events as an infectious illness spread, and they search the internet for disease-related terms (Chen et al., 2019; Shang et al., 2021). High-impact incidents, such as COVID-19, often get a lot of media attention, resulting in heightened public anxiety (Tulloch and Zinn, 2011; Young et al., 2013; Sun et al., 2021) and long-term repercussions (Tulloch and Zinn, 2011; Young et al., 2013). The connection between the media and financial markets has been well-documented in the literature for decades (e.g., Barberis et al., 1998; Tetlock, 2007). Akıncı and Chahrour (2018) and Cohen et al. (2018) have supported the so-called “bad news principle,” which states that only negative news affects investment choices. However, some writers argue that good news may equally influence investment decisions (Narayan and Bannigidadmath, 2015; Narayan, 2019). Technological advancements and the ease with which individuals may access the internet have resulted in the availability of a huge quantity of information that people can access on a daily basis. Several social phenomena have been tracked using web search traffic (Choi and Varian, 2012). People’s thinking has shifted as a result of mass online activity (Preis et al., 2012; Moat et al., 2014), and a new science of collective decision making has emerged. Online sources have been used as proxies in finance research to explain the collective behavior of financial markets (Heiberger, 2015; Nguyen et al., 2019; Tan and Taş, 2019). Several indicators were utilized, including Yahoo online search traffic (Bordino et al., 2012), Twitter activity (Zhang et al., 2011; Mathiesen et al., 2013), Google Trends (Kristoufek, 2013; Asif Khan et al., 2019; Nguyen et al., 2019), and Wikipedia activity (Nguyen et al., 2019). Google Trends’ explanatory power for the effect of COVID-induced public anxiety on stock market returns has been evaluated in a stress scenario. This issue has been addressed in recent literature. Costola et al. (2020) looked at the dynamics of public concern in Italy, the first European nation to suffer a COVID-19 epidemic, using three search-engine data from Google Trends. According to their research, public worry in Italy is a driver of public concern in other countries, and Google Trends data for Italy better explain stock index returns in other countries when compared to country-based variables. Similarly, Liu (2020) found that the negative COVID-19 effect on China’s composite index varied by industry based on daily Google Trends search data. Smales (2020) demonstrated how increased attention to COVID-19, as assessed by Google Search Volumes, impacted United States market returns adversely. Increased Google Trends synthetic index for COVID-19 has a direct and indirect impact on implied volatility and stock returns, according to Papadamou et al. (2020), with the effects being greater in Europe than the rest of the globe. Meanwhile, Wikipedia has not gotten the same amount of attention as Google Trends as a proxy for measuring investor anxiety, despite some evidence from the literature. Cergol and Omladič (2015) demonstrated that Wikipedia entries for stock prices exhibit an inverted pattern of a “merry frown” for the bear market and a “sour grin” for the bull market. Hervé et al. (2019) investigated the connection between Wikipedia records and the conduct of noise traders, finding that solely the attention of noise traders affects stock returns and increases volatility. After reviewing prior research and interpreting fear as a collective behavior arising from an outburst of public concern over an issue (Loewenstein et al., 2001), the following is suggested as the study’s initial hypothesis being put to the test.

H01: Public anxiety caused by COVID lowers European stock returns.

One key issue is whether COVID-induced public dread, in addition to being harmful, is also beneficial having the ability to explain stock market returns also means being able to forecast them in the future a wide range of options are available. There has been research on the predictive power of many internet sources, such as: Twitter sentiments (Bollen et al., 2011), stock micro-blog sentiments (Oh and Sheng, 2011), Google Trends (Preis et al., 2013; Bijl et al., 2016; Tan and Taş, 2019), and Wikipedia search volumes (Curme et al., 2014). Google Trends is one of the most widely used indicators, not only because of its explanatory power (Heiberger, 2015; Yu et al., 2019), but also because of its utility as a source of data for quantifying trading behavior (Preis et al., 2013), earnings announcements (Drake et al., 2012), and international equity holdings (Mondria et al., 2010). Changes in Wikipedia page views offered information for investors to make choices before stock trading, according to Moat et al. (2013) in finance. Weng et al. (2018) examined conventional stock technical indicators in combination with internet data sources (such as Google and Wikipedia) to forecast short-term stock prices using a large sample of firms from the S&P 500 Index. They demonstrated that using internet data sources improves stock price prediction in their research. The epidemic has given an excellent opportunity to review the predictive potential of online-based measures and put them to the test in a stressful situation. Salisu and Vo (2020) demonstrated the predictive potential of Google Trends by using health-news searches adjusted for macroeconomic variables and incorporating financial news in the prediction of stock returns, both using Google’s search engine. Lyócsa et al. (2020) used Google search traffic as a predictor of anxiety and dread words relating to the COVID-19 outbreak and non-pharmaceutical terms physical contagion intervention policies Their findings supported the belief that fear is a real thing the COVID-19’s product is an excellent predictor of stock market fluctuations. Wikimedia Commons there have been no studies based on Wikipedia search volumes that we are aware of stock market performance may be predicted using COVID-19. The study second hypothesis, based on the above, is as follows:

H02: Public panic caused by COVID may be used to forecast European stock returns.

Several researches have designed trading techniques based on the data discovered about the predictive potential of internet search traffic. We are aware that there is just a small body of research that looks into Google Trends and/or Wikipedia data in this context in particular. Curme et al. (2014) suggested trading methods based on the data created by internet users while looking for information online utilizing the Google search engine and the Wikipedia encyclopedia. They pointed out that search levels in the areas of politics and business were effective in foreseeing events in the stock markets subsequent moves, in particular, they demonstrated how these searches anticipated future market declines. According to Bijl et al. (2016), the high number of Google searches for publicly traded businesses results in negative returns stocks. As a result, these writers looked into a trading strategy that included selling high-yield equities. Purchasing equities with low Google search volume vs. buying stocks with high Google search volume. They showed how these techniques might provide positive returns even when transaction expenses were taken into account. Gómez et al. (2018) suggested the use of Google search volumes to build algorithmic trading systems based on different financial keywords. They discovered that the number of Google searches is a useful indicator for gauging worldwide investor sentiment in their research. Dickerson (2018) demonstrated a very successful algorithmic trading method for trading Bit-coin based on Wikipedia and Google search traffic. ElBahrawy et al. (2019) discovered that, when compared to baseline methods, a basic trading strategy based on Wikipedia’s opinions on digital currencies provided substantial returns on investment. The third hypothesis to be investigated, based on the literature, is as follows:

H03: A Google Trends and Wikipedia-based algorithmic trading system may provide significant advantages.

Based on the limitations in the research, this study will attempt to fill those gaps by assessing the explanatory and predictive capacity of COVID-induced public anxiety using Google Trends and Wikipedia as search engines. Furthermore, this research suggests incorporating COVID-19’s fear into the design of an algorithmic trading system. This is the only research that we are aware of that suggests a trading system based on COVID-induced public anxiety utilizing internet search levels as proxy for this concern.

## Research Methodology

A sample of daily data was collected from January 2, 2020 to September 17, 2020 in order to examine the impact of public anxiety created by COVID-19 on the recovery of European stock markets.

In this research, we suggested two indicators as proxy for Internet users’ behaviors to assess public dread. On the one hand, Google Trends^1^ (Fear1) internet search data was chosen using the same techniques as Chen et al. (2020) and Lyócsa et al. (2020). Google Trends provides a free and public query index that depicts search volume as a number between 0 and 1. Hundreds of categories may be used to categories data, such as geographic location activities, and so on. The “search by subject” option was selected for our research, and the topic “COVID-19” was chosen. Instead of an introduction, the COVID-19 subject contained questions about the virus precise phrase. This subject allows for a variety of synonyms, spelling mistakes, and grammatical blunders to be included in the study due to translations from other languages (Szmuda et al., 2020). In addition, Wikipedia search data^2^ (Fear2) was used additionally chosen. Researchers have looked at prediction models based on Wikipedia queries. Mestyán et al. (2013) and Moat et al. (2013) are examples of earlier authors (2013). Look up something on Wikipedia. Page views analysis has volumes accessible, and each extracted data set displays the total amount of data collected by users in a single day across the globe. Similarly, the subject of discussion was ‘‘COVID-19,’’ however, unlike Google, Wikipedia did not provide statistics by nation. Google Trends is a tool that allows you to see how COVID-induced public anxiety affects the European stock market. This research includes nations that are members of the European Union and have a population of more than one million people, a city with a population of more than ten million people and those with a thriving stock market and a representative index were chosen. As a result, for the nations who fulfilled these criteria and had sufficient stock, investing^3^ provided market data, including stock prices. Their main stock indexes were utilized in the analysis. Table 1 lists the nations and stock indexes that were chosen. They showed how these techniques might provide positive returns even when transaction expenses were taken into account. Gómez et al. (2018) suggested the use of Google search volumes to build algorithmic trading systems based on different financial keywords. They discovered that the number of Google searches is a useful indicator for gauging worldwide investor sentiment in their research. Dickerson (2018) demonstrated a very successful algorithmic trading method for trading Bit-coin based on Wikipedia and Google search traffic. ElBahrawy et al. (2019) discovered that, when compared to baseline methods, a basic trading strategy based on Wikipedia’s opinions on digital currencies provided substantial returns on investment. The third hypothesis to be investigated, based on the literature, is as follows: H03: A Google Trends and Wikipedia-based algorithmic trading system may provide significant advantages. Based on the limitations in the research, this study will attempt to fill those gaps by assessing the explanatory and predictive capacity of COVID-induced public anxiety using Google Trends and Wikipedia as search engines. Furthermore, this research suggests incorporating COVID-19’s fear into the design of an algorithmic trading system. This is the only research that we are aware of that suggests a trading system based on COVID-induced public anxiety utilizing internet search levels as proxy for this concern.

**TABLE 1:** Countries and stock indices taken as reference points.

Country	Stock index
Spain	IBEX 35
United Kingdom	FTSE 100
Greece	Athens General Composite
Poland	WIG 20
Germany	DAX 30
Romania	BET
Portugal	PSI 20
Netherlands	AEX
Turkey	BIST 100
France	CAC 40
Belgium	BEL 20
Italy	FTSE MIB

### Research Designs

We decided not to use the cointegration analysis since all variables were not integrated in the same order, as Enders (2004) suggested. To remove the unit root issue caused by the use of non-stationary variables, we suggested that differences are to be entered into non-stationary variables in this research to address the spurious regression problem (Montero, 2013). In this research, we suggested a panel data method with all stationary variables. When the data provided included a temporal and a transversal dimension, this method allowed for the assessment of the explanatory power of certain variables over others, making it a more suitable approach than transversal analysis and time series analysis in this situation (Wooldridge, 2011). The fixed effects model and the random effects model are the two primary models used in panel data technique. The individual impact is assumed to be associated with the explanatory variables in the fixed effects model. The random effects model, on the other hand, implies that individual effects are unrelated to the model’s explanatory factors. The use of the Hausman test (Hausman, 1978) was critical in determining which model was most suitable. This test determined if the panel data model’s determinants were more consistent whether using the fixed effects model *versus* the random effects model. The findings of the Hausman test indicated whether or not the random effects model should be used (Tables 2, 3). As a result, the following panel data models were suggested to investigate the impact of COVID-induced public anxiety on European stock market returns:


(1) $$\begin{array}{c} R_{1it}=\alpha +\beta_{1}d.Fear_{1it}+\beta_{2}Gold_{\text{it}}+\beta_{3}VIX_{\text{it}}+\beta_{4}TV_{\text{it}}\\ \;+w_{\text{i}}+\varepsilon_{\text{it}},t=1,2,\ldots ,T \end{array}$$

(2) $$\begin{array}{c} R_{1it}=\alpha +\beta_{1}d.Fear_{2it}+\beta_{2}Gold_{\text{it}}+\beta_{3}VIX_{\text{it}}+\beta_{4}TV_{\text{it}}\\ \;+w_{\text{i}}+\varepsilon_{\text{it}},t=1,2,\ldots ,T \end{array}$$

(3) $$\begin{array}{c} R_{2it}=\alpha +\beta_{1}d.Fear_{1it}+\beta_{2}Gold_{\text{it}}+\beta_{3}VIX_{\text{it}}+\beta_{4}TV_{\text{it}}\\ \;+w_{\text{i}}+\varepsilon_{\text{it}},t=1,2,\ldots ,T \end{array}$$

(4) $$\begin{array}{c} R_{2it}=\alpha +\beta_{1}d.Fear_{2it}+\beta_{2}Gold_{\text{it}}+\beta_{3}VIX_{\text{it}}+\beta_{4}TV_{\text{it}}\\ \;+w_{\text{i}}+\varepsilon_{\text{it}},t=1,2,\ldots ,T \end{array}$$

Where R_1it_ and R_2it_ are the dependent variables of the models, α is the constant term, β_k_ is the regression coefficient of each explanatory variable k, w_i_ is a random variable of the individual effects, and ε_it_ is the error term.

**TABLE 2:** Influence of COVID-induced public fear on European stock market returns.

Variable	R_1i_				R_1i_			
	Coef.	S.D.	*Z*	*p*-value	Coef.	S.D.	*z*	*p*-value
Const	–0.00035	0.00035	−3.543	0.305	−0.00035	0.00037	−3.490	0.335
d.Fear3t	–0.00033	3.35e-5	−9.737	0.000***				
d.Fear3t					−7.95e-5	0.00030	−0.408	0.583
Gold_t_	0.08953	0.02438	7.937	0.000***	0.30393	0.03340	9.330	0.000***
VIX_t_	–0.04395	0.00355	−38.42	0.000***	−0.04530	0.00357	−39.50	0.000***
TV_t_	0.00033	0.00073	0.58	0.495	−0.00073	0.00038	−0.353	0.567
Durbin Watson test			3.35455				3.43837	
Hausman			0.57053				0.44539	
Test			−0.955				−0.864	
Obs.			3,690				3,690	

***indicate the significance at 3, 5, and 30% levels, respectively.

**TABLE 3:** Influence of COVID-induced public fear on European stock market returns.

Variable	R2i				R2i			
	Coef.	S.D.	*Z*	*p*-value	Coef.	S.D.	*z*	*p*-value
Const	–0.00034	0.00016	−1.631	0.105	−0.00025	0.00017	−1.490	0.136
d.Fear_1t_	–0.00047	3.34e-5	−9.717	0.000***				
d.Fear_2t_					−7.95e-5	0.00020	−0.408	0.821
Gold_t_	0.08951	0.02478	7.937	0.000***	0.10383	0.02340	9.120	0.000***
VIX_t_	–0.04386	0.00373	−38.33	0.000***	−0.04620	0.00367	−29.50	0.000***
TV_t_	0.00031	0.00047	0.72	0.495	−0.00012	0.00047	−0.362	0.717
Durbin Watson test			2.36445				2.13838	
Hausman			0.63083				0.48979	
Test			−0.875				−0.885	
Obs.			3,690				3,690	

***indicate the significance at 1, 5, and 10% levels, respectively.

### Data Collection Method

A sample of daily data was collected from January 2, 2020 to September 17, 2020 in order to examine the impact of public anxiety created by COVID-19 on the recovery of European stock markets. In this research, we suggested two indicators as proxy for Internet users’ behaviors to assess public dread. On the one hand, Google Trends (see text footnote 1) (Fear1) internet search data was chosen using the same techniques as suggested by Chen et al. (2020) and Lyócsa et al. (2020). Google Trends provides a free and public query index that depicts search volume as a number between 0 and 1. Hundreds of categories may be used to categorize data, such as geographic location activities, and so the “search by subject” option was selected for our research, and the topic “COVID-19” was chosen. Instead of an introduction, the COVID-19 subject contained questions about the virus precise phrase this subject allows for a variety of synonyms, spelling mistakes, and grammatical blunders. To be included in the study are translations from other languages (Szmuda et al., 2020). In addition, Wikipedia search data (see text footnote 2) (Fear2) was used additionally chosen Researchers have looked at prediction models based on Wikipedia queries. Mestyán et al. (2013) and Moat et al. (2013) are examples of earlier authors (2013). Look up something on Wikipedia. Page views Analysis has volumes accessible, and each extracted data set displays the total amount of data collected by users in a single day across the globe. Similarly, the subject of discussion was “COVID-19,” however, unlike Google, Wikipedia did not provide statistics by nation. Google Trends is a tool that allows you to see how COVID-induced public anxiety affects the European stock market. This research includes nations that are members of the European Union and have a population of more than one million people, a city with a population of more than ten million people and those with a thriving stock market and a representative index were chosen. As a result, for the nations who fulfilled these criteria and had sufficient stock, investing (see text footnote 3) provided market data, including stock prices. Their main stock indexes were utilized in the analysis. Table 1 lists the nations and stock indexes that were chosen.

To achieve better soundness in the obtained findings, we chose two variables of stock index returns to assess stock returns. The rate of change in the closing price of a stock index over 2 days was the first metric utilized.


(5) $$S_{1it}=\frac{S_{it}-S_{i,t-1}}{S_{i,t-1}}$$

Where *S*_1*it*_ is the return of the stock index *i* on day *t* as calculated using eq. (5), and *S*_*it*_ is the index’s closing price on day *t*. The logarithm of the ratio of the closing prices over two consecutive days was the second measure of stock returns.


(6) $$S_{2it}=\log\left(\frac{S_{it}}{S_{i,t-1}}\right)$$

Where *S*_2*it*_ is the return of index I on day t, calculated using eq. (6), and control factors linked to the COVID-19’s effect on stock market results were also examined. Gold returns (Cepoi, 2020; Gherghina et al., 2020), the rate of change of the volatility index (VIX) (Liu et al., 2020; Salisu et al., 2020b), and the rate of change of trading volume (TV) were utilized as control variables in this research (Huo and Qiu, 2020; Zaremba et al., 2020). The descriptive statistics for the variables under investigation are shown in Table 4. In terms of stock return metrics, the findings showed that throughout the research period, the average daily return was negative, with the lowest return occurring on March 12, 2020, when the Italian market saw a price drop of −16.924%. According to Google Trends, the greatest COVID-induced public panic occurred between March 11 and 18, whereas the highest Wikipedia search traffic occurred on January 27. Using the control variables as a guide, the findings revealed that gold maintained a positive return throughout the study period, reaching a high of 5.77% on March 23.

**TABLE 4:** The targeted variables’ descriptive statistics.

Variable	Mean	Median	Minimum	Maximum	S.D.	C.V
R_1_	−0.00097	0.00069	−0.16914	0.10976	0.01149	14.569
R1	−0.00049	0.00027	−0.09061	0.04616	0.00649	19.701
Fear_1_	16.77	10	0	100	17.491	1.1091
Fear1	2.17E + 05	19,861	907	9.92E + 05	1.95E + 05	1.4976
Gold	0.0016	0.0016	−0.0469	0.0677	0.01417	10.961
VIX	0.00764	−0.011	−0.1667	0.4796	0.10499	16.764
TV	0.06996	−0.00476	−0.91591	9.9165	0.60436	7.134

*Critical value at 6% (two-tailed) = 0.0400.*

The VIX reached its highest rate of change of 47.87% on June 11, 2020, while the Romanian market saw its maximum and lowest rate of change of TV on February 24 to August 18, 2020, respectively. When looking at the dispersion, it is clear that stock returns have the greatest dispersion of the variables examined. The relationships between the various target research variables are shown in Table 5. It demonstrates how the suggested return measures were negatively associated with COVID-induced public anxiety (both Fear1 and Fear2), the VIX index, and TVs, whereas gold returns were favorably correlated. Concerning the representative measures of COVID-induced public anxiety, it is discovered that, as one would expect, both measures are positively linked. These indicators also have a negative relationship with gold returns and TVs, but a positive relationship with the VIX index.

**TABLE 5:** Results of bivariate correlations of the targeted variables.

Variable	R_1_	R7	Fear_1_	Fear7	Gold	VIX	TV
R_1_	1	0.1474	–0.1607	–0.1573	0.1704	–0.4147	–0.0734
R7		1	–0.154	–0.1476	0.171	–0.4704	–0.066
Fear_1_			1	0.4447	–0.1164	0.0634	–0.0053
Fear7				1	–0.0477	0.7044	0.0073
Gold					1	–0.0457	0.0047
VIX						1	0.1471
TV							1

*Critical value at 5% (two-tailed) = 0.0400.*

## Results and Discussion

The results obtained in the study with the proposed models. Table 2 shows the findings of a study that looked at the effect of COVID-induced public dread (d.Fear_1t_ and d.Fear_2t_) on European stock market returns, taking the rate of change of the market price into account (R_1t_). With over 99.9% confidence, COVID-induced public anxiety, as assessed by Google Trends, was shown to have a negative and substantial effect on European stock indexes’ returns. When the control factors were taken into account, gold returns had a positive and substantial effect on European stock market returns, whereas the VIX index variation had a negative and significant impact. TVs, on the other hand, had no discernible impact. Although the coefficient of COVID-induced public anxiety assessed by Wikipedia searches (d.Fear_2t_) had a negative value, it cannot be stated that the coefficient had a value different from 0.

The restriction of Wikipedia’s search level extraction may be one of the explanations for this finding. It was not feasible to extract the level of searches for the COVID-19 subject by nation, as stated in the data section. Only global detail was available on the platform. As a result, although COVID-induced public dread may vary by nation, the limits of the global metric based on Wikipedia may not properly represent COVID-19-related fear in a given country’s population. When we look at the findings for the models suggested around the second measure of the return, R_2t_ (Table 3), we see that they are comparable to those in Table 2. The models satisfy the residuals’ uncorrelated and homoscedasticity in both instances, demonstrating how COVID-induced public anxiety, as assessed by Google search volumes, has a negative and substantial effect on European stock market returns. Similarly, it was discovered that the reference based on Wikipedia search volumes had no meaningful impact on stock market results. H01 can be accepted based on the results obtained when evaluating the impact of COVID-induced public fear on European stock market returns using d.Fear_2t_, which show that increases in fear caused by the COVID-19 resulted in lower European stock market returns. These findings matched with Costola et al. (2020), Liu (2020), Papadamou et al. (2020), and Smales (2020) specification of the variables, which was modified to check the robustness of the analysis, taking into account the lagged ones to validate the results.

We looked at the effects of the explanatory variables at time t-1 on stock market returns at time t in particular. As a result, this specification not only assesses the robustness of our findings, but also the ability of COVID-induced public fear to predict next-day stock market returns. Table 6 shows the results of applying a lag to the proposed models’ explanatory variables. When using Google Trends as a reference, it can be seen that even after applying a lag, COVID-induced public fear continues to have a significant inverse relationship with stock market returns with more than 99.9% confidence. When it came to Wikipedia search volumes, however, there was no discernible influence. Similarly, the variables of gold returns and VIX index variation maintained the relationship established in previous models. As a result, these findings show that COVID-induced public fear not only explains but also predicts the return of European stock markets, allowing H02 to be accepted. The validation of this research hypothesis backs up Lyócsa et al. (2020) and Salisu and Vo (2020) conclusions about this metric’s predictive power. This research aims to take things a step further by using the findings so far. After demonstrating that COVID-induced public fear explains and forecasts a portion of the performance of European stock markets, we looked into the potential of making huge gains by investing in these markets based on COVID-induced public fear.

**TABLE 6:** The predictive capacity of the COVID-induced public fear of the European stock markets 24 return.

Variable	*z*	*Z*	*Z*	*Z*
	Coef. (*p-*value)	Coef. (*p-*value)	Coef. (*p-*value)	Coef. (*p-*value)
Const	−2.01**	−1.84*	−2.52**	−2.34**
	–0.0009	–0.0008	–0.0005	–0.0005
	–0.044	–0.066	–0.012	–0.019
d.Fear_1t–1_	−8.69***		−8.75***	
	–0.0008		–0.0003	
	0		0	
d.Fear_2t–1_		–0.75		–0.71
		–0.0004		–0.0002
		–0.452		–0.477
Gold_t–1_	2.96***	4.07***	2.90***	4.02***
	0.0901	0.1247	0.039	0.0544
	–0.003	0	–0.004	0
VIX_t–1_	−3.68***	−4.93***	–3.72	−4.97***
	–0.0154	–0.0207	–0.0069	–0.0092
	0	0	0	0
TVt-1	0.58	–0.3	0.67	–0.27
	0.0005	–0.0003	0.0003	–0.0001
	–0.564	–0.719	–0.502	–0.785
Durbin Watson	2.31524	2.22535	2.31838	2.22512
Hausman test	0.4119	0.3318	0.4487	0.3868
Test	–0.982	–0.988	–0.978	–0.98355
Obs.	3,690	3,690	3,690	3,690

***, **, and * indicate the significance at 1, 5, and 10% levels, respectively.

The use of Wikipedia search volumes was ruled out owing to the absence of correlation between Wikipedia search volumes and stock returns. As a result, the initial model suggested used Google search volumes (Eq. 7) in conjunction with the lag of explanatory factors whose effect on stock market returns was shown to be substantial. An algorithmic trading system was utilized in the suggested system architecture to make investment choices based on the following equation:


(7) $$\begin{array}{c} R_{1it}=-0.000847037-0.000756169\cdot d.Fear_{1it-1}\\ \;+0.0905054\cdot Gold_{\mathrm{it}-1}-0.0150954\cdot VIX_{\mathrm{it}-1} \end{array}$$

Using the preceding equation as a guide, we created an algorithmic trading system that worked as follows:

• If the equation’s result was positive, open a long position in the index’s future at the start.
• If the equation’s outcome was negative, open a short position in the index’s future.
• At the conclusion of the trading session, all open positions were closed.

We used the Trading Motion platform^4^ to conduct these back tests. The Trading Motion has been providing automated trading systems from various developers to clients *via* more than 20 brokers across the globe since 2002.

**FIGURE 1:**
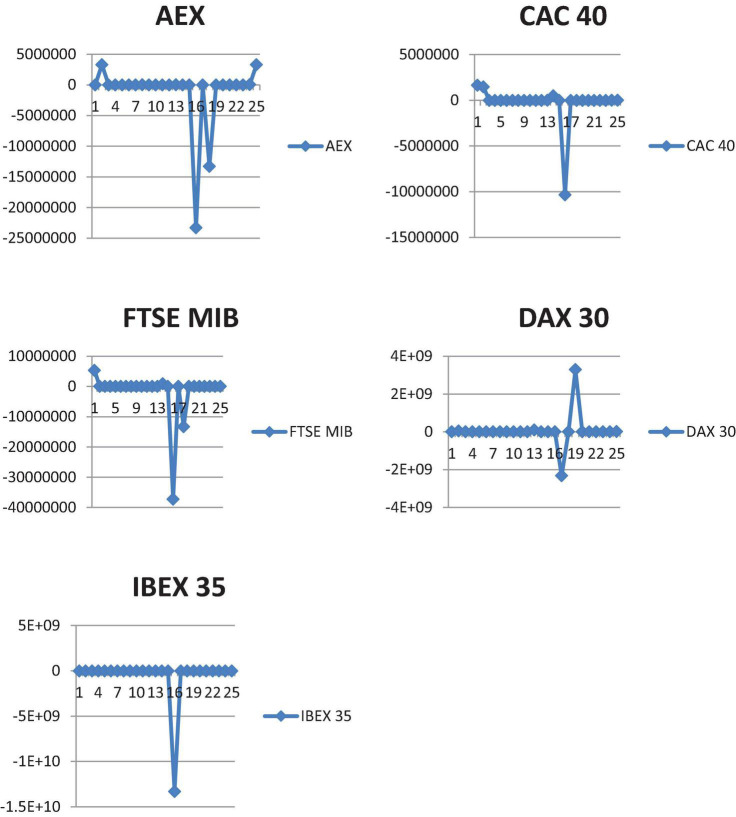
Algorithmic trading system P&L for AEX, CAC 40, DAX 30, IBEX 35, and FTSE MIB.

In terms of the European market, this platform enables you to trade the following indices: AEX, CAC 40, DAX 30, IBEX 35, and FTSE MIB. As a consequence, we examined the outcomes of applying the suggested model to these five indices. Figure 1 shows the Profit and Loss (P and L) graphs produced after the specified procedure. The trading algorithms beat the market for all indices throughout the research period and were profitable during the epidemic phase that corresponded with the large lockdowns. However, it was discovered that the system’s performance suffered as a consequence of the return to normalcy.

**FIGURE 2:**
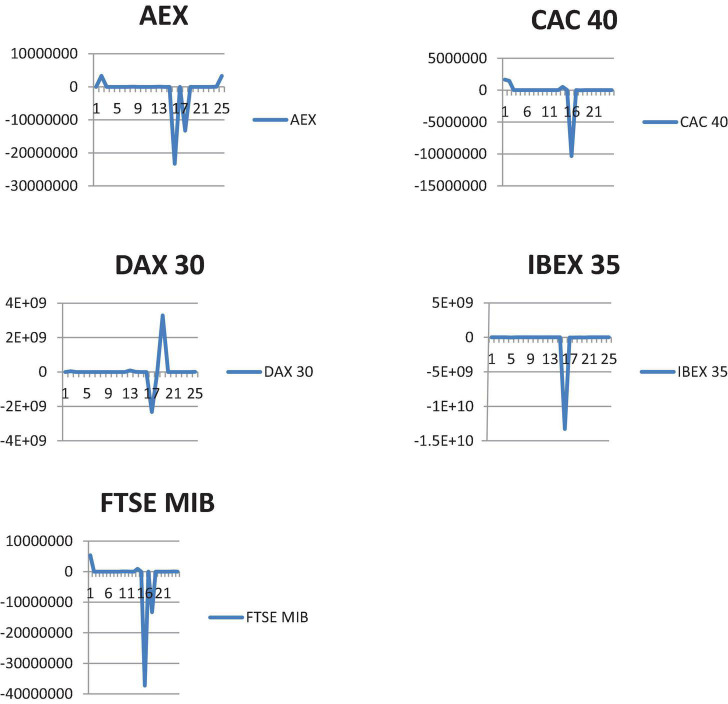
Optimized algorithmic trading system P&L for AEX, CAC 40, DAX 30, IBEX, and FTSE MIB.

As a result, the system needed to be optimized. We only examined two criteria to prevent over-optimization: closing positions when a profit of 1% was reached and the system only operating when the equation produced a result higher than 0.25%. Figure 2 shows the P and L charts of the optimized systems. It can be shown that using the optimized trading system resulted in improved performance not only just during the pandemic’s containment phase, which corresponded with large lockdowns, but also after the closures were lifted. Table 7 shows the performance of the improved algorithmic trading system. We can observe that the five optimized systems had a positive gross and net return. The profit factor was higher than one, and the proportion of winning sessions was greater than 50%, both of which are considered lucrative levels. As a result, all of the annualized return on investments (ROIs) in Table 7 are positive. The ROI was determined by dividing the net P and L by the capital positions recommended by stockbrokers.

**TABLE 7:** Summary of the improved trading system performance.

Indicator	AEX	CAC 40	DAX 30	IBEX 35	FTSE MIB
Net P&L	2929.5329	1655037.29	59215.15	5150	5345037.1
Gross P&L	3290290	1465400	42536505.5	5050	5370
Profit factor	1.1346	1.1329	2.05	1.29	1.19
Sharpe ratio	0.29	0.29	2.95	1.59	0.5329
Slippage per side	−0.05	−0.29	−1.1329	−1	−0.05
Net P&L over	5.03%	5.95%	29.92%	32903290.33%	11.29%
Drawdown	0.37	0.29	5.59	1.51	0.32901
Mathematical expectation	34509.54	19.52	345.5	43.37	59.25
Analyzed sessions	151	190	159	190	151
Sessions in market	29	29	29	29	53290
Winning sessions	25	29	32905	25	32900
Success rate	29.29%	53290.05%	29.52%	52.37%	29.50%
Winning sessions profit	37909.51	11500.09	95034305.29	19250	25110.29
Average losing sessions	929.1329	503290.29	3793290.29	295.21	903290.59
Losing profit sessions	20	15	20	15	23290
Sessions profit	−23290491.99	−10329029.53	−29295.52	−13290132900	−37293290.29
Average worst sessions	−1152.5	−510.29	−2329053291	−292.32905	−990.59
Drawdown best sessions	−13290537.09	−2950.01	−11025.29	−5329050	−13290295.52
Worst sessions	1195	295.91	3290329026	932900	1195
	−3795.52	−1291.25	−11025.29	−2150	−3715.52
30 days volatility	132905.29%	50.37%	32900.52%	29.90%	95.95%
1 year volatility	0.00%	0.00%	0.00%	0.00%	0.00%
5 years volatility	0.00%	0.00%	0.00%	0.00%	0.00%
Suggested capital	52000	19000	120000	25000	50000
Required capital	3290500	2000	13290500	2500	2500

The results support H03 by showing that exceptional gains may be obtained *via* algorithmic trading systems based on COVID-induced public anxiety. Previous studies such as Curme et al. (2014), Bijl et al. (2016), Dickerson (2018), and Gómez et al. (2018) found good performance of trading systems based on the level of Google searches, and this study adds new evidence of the possibility of considering COVID-induced public fear in the construction of algorithmic trading systems.

## Conclusion

The announcement of the COVID-19 pandemic was the most important worldwide shift since the turn of the century. Stock markets fell sharply as a result of the pandemic, with the most severe drops in the history of world financial markets occurring in February and March 2020. Because of the effect of COVID-19’s ramifications on stock prices, this research was conducted. We examined the effect of COVID-induced public anxiety on European stock markets in markets here are market returns. In accordance with prior research, when a population feels frightened, they become more aggressive. They are more likely to seek knowledge about the incident that causes them to feel this way and regarded COVID-19 search volumes on Google and Wikipedia as adequate. COVID-induced public anxiety is represented by proxies from January 2, panel data analysis was used on a sample of 13 European nations. We demonstrated that COVID-induced public anxiety, from September 17, 2020 to September 17, 2020, had a negative and substantial effect on Google search volumes, as assessed by Google search volumes. Returns on European stock exchanges using the number of searches on Wikipedia as a proxy for fear COVID-19, number, was determined to have no effect. These findings were unaffected by the addition of a lag in the explanatory factors, showing the explanatory variables’ predictive ability models that have been suggested, and another significant aim stated in this research was the goal of obtaining exceptional gains from algorithmic trading based on COVID-induced public anxiety systems. The ability of internet searches to explain and forecast has been extensively discussed, and the searches have been investigated, although there is scant evidence of their use in algorithmic trading systems, particularly under stressful situations like the current COVID-19 epidemic. As a result, taking into account the suggested model with Google’s delayed explanatory factors, the important factors include 18 COVID-19 search volumes, gold returns, and the VIX index. Algorithmic trading methods based on COVID-induced public anxiety were used in the model (19). AEX, CAC 40, DAX 30, IBEX 35, and AEX were created for European stock market indexes, FTSE MIB. The findings revealed the potential of making a huge profit using then the basis of COVID-induced public anxiety, and 24 models have been suggested. It discovered how these systems work, provided good gross and net returns, a positive profit factor, and a% age of profit there have been 28 trading sessions that have resulted in a profit of more than 50%. The results of this research have a number of significant ramifications. On the one hand, the fact that public anxiety generated by COVID has a negative and substantial effect on the significance of this concern for the effective functioning of European stock markets of these markets’ performance. As a result, the population’s fear of the unknown must be managed by the government and the media must pay attention to the COVID-19 epidemic. This emphasizes the importance of the significance of finding the appropriate balance between the necessary concern from a health standpoint and point of view, as well as the possibility of a panic attack with severe consequences in the economy and, in particular, in stock markets. Furthermore, the data suggested that exceptional gains might be obtained, which are helpful for using algorithmic trading systems based on COVID-induced public anxiety investors, giving them the opportunity to profit from their investments. In the context of financial uncertainty resulting from the health crisis, 50 equity investments were made. Finally, we propose that further research paths based on the observed findings offer a far more in-depth knowledge of how illness fear affects stock prices from both a theoretical and methodological standpoint. Similarly, the outcomes achieved with the suggested algorithmic trading systems, significant research in the field is encouraged explanation of effective investing methods under adversity, such as a real-life financial crisis pandemic. Furthermore, although independent stock indexes were taken into account in this study, it would be interesting to take into account the public’s fear of infectious diseases building diverse investment portfolios, which is number six on the list.

## Data Availability Statement

The original contributions presented in the study are included in the article/supplementary material, further inquiries can be directed to the corresponding author/s.

## Ethics Statement

Ethical review and approval was not required for the study on human participants in accordance with the Local Legislation and Institutional Requirements. Written informed consent for participation was not required for this study in accordance with the National Legislation and the Institutional Requirements.

## Author Contributions

YS: conceptualization, methodology, supervision, and writing – reviewing and editing. HL: conceptualization, software, formal analysis, and writing – reviewing and editing. YC: conceptualization, methodology, and writing – original draft. All authors contributed to the article and approved the submitted version.

## Conflict of Interest

The authors declare that the research was conducted in the absence of any commercial or financial relationships that could be construed as a potential conflict of interest.

## Publisher’s Note

All claims expressed in this article are solely those of the authors and do not necessarily represent those of their affiliated organizations, or those of the publisher, the editors and the reviewers. Any product that may be evaluated in this article, or claim that may be made by its manufacturer, is not guaranteed or endorsed by the publisher.

## Footnotes

1
https://trends.google.com/trends/

2
https://pageviews.toolforge.org/

3
https://es.investing.com/

4
https://www.tradingmotion.com/
